# Editorial: Multimodality imaging in cardiomyopathy

**DOI:** 10.3389/fcvm.2022.1082023

**Published:** 2023-01-11

**Authors:** Allison G. Hays, Andrew D. Choi, Juan Lopez-Mattei, Monica Mukherjee

**Affiliations:** ^1^Division of Cardiology, Johns Hopkins University School of Medicine, Baltimore, MD, United States; ^2^Division of Cardiology, Department of Radiology, The George Washington University School of Medicine, Washington, DC, United States; ^3^Heart and Vascular Institute, Lee Health Hospital, Fort Myers, FL, United States

**Keywords:** echocardiography, cardiac magnetic resonance (CMR) imaging, cardiac computed tomographic (CT) imaging, positron emission tomography (PET), multimodality imaging

To the Editor,

*Ad astra per aspera (“Through adversity, to the stars”)—Adapted from Virgil*

The recent coronavirus-19 (COVID-19) pandemic brought global society to a standstill, leading to a transformation across medicine and the retooling of cardiovascular medicine to adopt evidence based best practices that prioritize safety, efficacy and outcomes (1). The practice of cardiovascular imaging was truly transformed in a multitude of ways across North America and the world, especially when stratified by modality (2, 3). Through this adversity, multimodality cardiovascular imaging remained at the forefront of cardiovascular care by allowing for new approaches in the assessment of cardiac structure and function, providing mechanistic insights into pathobiology of disease. Cardiomyopathies vary widely in phenotype and clinical expression, as well as variable genetic underpinnings vs. those resulting from acquired states. Non-invasive imaging modalities such as echocardiography, cardiac CT (CCT), cardiac MRI (CMR), and nuclear techniques including positron emission tomography (PET) and SPECT imaging allow for delineation of cardiomyopathy phenotype as well as facilitating earlier diagnosis, risk stratification, and guidance of therapeutic decisions in cardiomyopathic states.

Given the broad range of techniques available, variability in utilization, and a range of expertise, the focus of this topic through state-of-the-art review articles (Covas et al.; Farrell et al.; Gambahaya et al.; Glynn et al.; Heidari-Bateni et al.; Ismail et al.; Scheel et al.; Wand et al.; Zghyer et al.) and selected original research (4) (Bi et al.; Sperry et al.; Tian et al.) was to survey contemporary clinical application of multimodality imaging techniques in advancing early diagnosis and management of patients with cardiomyopathies. We presented several review articles discussing common cardiomyopathies, specifically addressing the utility of multimodality imaging methods in early diagnosis, serial monitoring of therapeutic efficacy, and longitudinal follow-up. We also focused on the histopathology of each cardiomyopathic state and how imaging can unmask mechanism. In addition, this Research Topic highlighted the role and clinical utility of measuring more subtle indices of myocardial function including speckle tracking strain with echocardiography and feature tracking techniques using CMR (Tian et al.). Several reviews also described the emerging role of myocardial mapping using CMR to quantify left ventricular fibrosis, inflammation and edema and nuclear PET, which can yield important information in the workup of inflammatory cardiomyopathies. We also included original work about the value of CMR in cancer patients with presumed cardiomyopathy (Heidari-Bateni et al.). As clinical research has rapidly evolved in the diagnosis and workup of stress (Zghyer et al.) and infiltrative cardiomyopathy such as cardiac sarcoidosis (Wand et al.) and cardiac amyloidosis (Scheel et al.), and cardiac manifestations from cancer (Heidari-Bateni et al.), pulmonary hypertension (Farrell et al.), HIV (Gambahaya et al.), pregnancy (Ismail et al.), and systemic diseases such as scleroderma (Glynn et al.) these state-of the-art reviews provide clinically applicable approaches to the diagnostic workup as well as the clinical value of each imaging modality. Further, artificial intelligence has erupted over the past decade through the use of machine learning algorithms (5). To address this, also included in this series, Covas et al. presented a review of recent advances in artificial intelligence in coronary artery disease imaging through algorithms that mimic human neural networks to improve cardiovascular risk prediction, accuracy identify coronary artery stenosis and allow for rapid evaluation of ischemia, flow and atherosclerosis quantification. These reviews present new frontiers in prevention, precision and monitoring of disease activity across many clinically relevant cardiomyopathic states, thereby providing practical guidance to clinicians across these conditions. Finally, these reviews provide a critical, balanced approach to the workup of cardiomyopathy, discussing the relative merits and weaknesses of each imaging technique.

Our series also included several important original research articles. In a pilotstudy by Sperry et al. evaluated the performance of F18-florbetapir as a novel tracer to identify ATTR cardiac amyloidosis. While data was limited by small sample size, it presents the tip of the iceberg in new frontiers in molecular PET imaging of amyloidosis. In a separate study, Tian et al. demonstrated the superiority of 3D right ventricular free wall strain over 2D free wall strain and conventional echocardiographic functional parameters in identifying the ischemic and non-ischemic etiologies of end-stage heart failure. This study importantly identifies the role of non-invasive imaging as a diagnostic tool to differentiate underlying cardiomyopathic etiologies.

To that end, these novel multimodality technologies hold promise for earlier diagnosis and non-invasive monitoring of cardiac involvement in systemic inflammatory diseases that will aid in preclinical studies, enhance patient selection, and provide surrogate end points in clinical trials, and improve clinical outcomes. In this post-pandemic era, *ad astra per aspera*, we hope this Research Topic provides new insights into clinical practice and allows practicing clinicians, trainees, and the global cardiovascular community to apply these advances in cardiovascular multimodality imaging practice.

## Author contributions

All authors listed have made a substantial, direct, and intellectual contribution to the work and approved it for publication.
